# Ulcerations of the Inguinal Glands and Penis, Relieved by the Chlorate of Lime

**Published:** 1829-01-01

**Authors:** 


### ST. BARTHOLOMEW'S HOSPITAL.

#### I. ULCERATIONS OF THE INGUINAL GLANDS AND PENIS, RELIEVED BY THE CHLORATE OF LIME.

A man was admitted under the care of Mr. Lawrence, on the 9th of July, presenting the following appearances. The penis was indurated throughout its whole extent, and covered with ill-conditioned sores. In the left groin was an ulcerated bubo, the edges of which were thickened and hard, whilst the centre contained a curd-like matter. On the right side was a large irregular swelling, dark in colour, and ulcerated in its centre. The sore was round and hollow, about the size of a shilling, and containing at its bottom the curdy substance existing in the bubo of the other side. The history given by the patient was as follows. He had suffered from gonorrhœa five or six times, and, two years before his admission, had a violent attack, attended with much inflammation and phymosis. The latter ran, indeed, to such a height, that the prepuce was divided, when those ulcers were found upon the glans, which remained at the time of his admission, and had never disappeared. The buboes in the groin were of shorter duration, and had burst of themselves.

He was ordered bread poultice, with the liquor opii sedativus to the sores, and took thirty minims of the tincture of opium night and morning, under which treatment, the irritable state, both local and general, was calmed. He was ordered, on the 11th, five grains of the compound extract of colocynth at bedtime, and the solution of the chloruret of lime to the parts. The health, on the 15th, was improved, and the ulcers perceptibly changing their character. *Essen. sarsæ*, ℥ss. *ter die*. In the course of a very few days, the character of the sore was completely changed—it looked comparatively healthy, was beginning to put on a healing edge, and lost the curdy matter from its surface. The penis was also not so hard, and the patient, in the course of some days, left the house, thinking he was well enough to return home.—*Gaz.*

The above case proves that rest, and appropriate remedial means, will frequently exert a salutary influence, even on diseases of malignant aspect. If the above was really an instance of scirrhus, it was certainly relieved, though a cure would, of course be hopeless, or nearly so. With regard to the "chloruret" of lime, we have often seen it tried, and cannot but think that its powers are, in general, greatly overrated. In sloughing sores and stumps, it can bear no comparison with the compound tincture of benzoin, and where it is used to correct or destroy the fetor of discharge, the *composite* odour produced, is infinitely worse, to some noses, than the original smell.

II. All must know how fatal extravasation of urine, from rupture of the urethra, was wont to be. In the present day, however, recoveries are not only common, but far the greater number of cases do well. In the olden time, 'tis scarce some twenty years ago, the practice was, to be sparing of the lancet, and profuse of bark—to make a few *punctures* in the cellular membrane, with the hope of letting off the extravasated fluid. In the present day, this practice is totally exploded, and a trifling puncture has now given way to numerous and bold scarifications, a practice alike scientific and successful.

*Case.* A sailor was received into Bartholomew's Hospital, with immense distention of the scrotum and penis, and great inflammation of the neighbouring parts, from rupture of the urethra and extravasation of urine, in consequence of an old and obstinate stricture. The rupture would appear to have occurred two days before, and in this condition the man had walked very nearly a mile to the hospital!

Mr. Earle, at his visit, made four or five incisions in the scrotum, and likewise cut down to the urethra behind the stricture, when a large flow of urine took place. Fomentations were applied, three grains of submuriate of mercury with one of pulvis opii given forthwith, and a saline mixture every four hours. The inflammation of the scrotum, and neighbouring parts, was relieved, the cellular membrane came away in the form of a slough, and, Nov. 10th, three weeks from his admission, the wound was doing well. At this time, the water chiefly came away by the natural channel, although no instrument could be passed through the stricture.—*Gaz.*

A case of a similar kind lately occurred at St. George's Hospital. Free scarifications in the scrotum, the penis, the pubes, and groins were made by Mr. Brodie, who, likewise, cut down upon the stricture, and tried, but ineffectually, to introduce an instrument thence into the bladder. Free suppuration took place in the cellular membrane, but sloughing was prevented. The patient was affected for the first few days with rather untoward and typhoid symptoms, but subsequently rallied. The treatment consisted in salines with laudanum, and afterwards bark.

